# School Climate, Peer Relationships, and Adolescent Mental Health: A Social Ecological Perspective

**DOI:** 10.1177/0044118X20970232

**Published:** 2020-11-04

**Authors:** Emily Long, Claudia Zucca, Helen Sweeting

**Affiliations:** 1 University of Glasgow, Glasgow, UK

**Keywords:** adolescents, mental health, peers, school climate

## Abstract

The current study investigated peer relationship and school climate factors associated with adolescent mental health. Cross-sectional data from 2,571 fifteen-year old students in 22 Scottish secondary schools was used. Multilevel models tested for school differences in mental health, and nested linear regression models estimated peer and school effects. Results demonstrated no significant between-school variation in mental health. Peer victimization was the only peer effect associated with mental health. School-belonging, student-teacher relationships, and a perceived inclusive school climate were associated with better mental health, whereas a perceived school climate of exam pressure was associated with worse mental health. The findings highlight multiple aspects of school climate that could be targeted in school-based interventions for adolescent mental health.

## Introduction

Mental health problems represent the largest single cause of disability within the UK (Mental Health Taskforce, 2016). Approximately half of all mental health disorders first emerge before age 14 (World Health Organization, 2018), and global estimates indicate the prevalence of mental health difficulties to be between 10% and 20% during adolescence. Moreover, adolescent mental health often shows a continued trajectory into adulthood (Bevilacqua et al., 2017; Gilbey et al., 2019; Shore et al., 2017), with a range of social and health consequences (Clayborne et al., 2019; Ohrnberger et al., 2017). As such, adolescent mental health has become a critical area for public health policy (House of Commons, 2019; World Health Organization, 2013), highlighting the need for research to uncover potentially modifiable factors associated with mental health during this life stage.

Given the large amount of time that adolescents spend within a school setting, schools have been increasingly recognized as a key context for implementing intervention efforts (Department of Health and Social Care and the Department of Education, 2017; Stigler et al., 2011). Viewed from a social ecological perspective (Sallis et al., 2008; Stokols, 1996), individual health outcomes (e.g., mental health) are the product of multiple, interacting systems of influence. Social ecological theory provides a framework for organizing risk and protective factors into a continuum of micro-, meso- and macro-levels, representing a range of individual, social, and community or environmental influences. During adolescence, these layers of influence include individual characteristics, family dynamics, peer relationships, and the wider environment, such as school context. By simultaneously considering multiple sources of influence, social ecological models thus provide contextualized insight into adolescent development. Importantly, whereas many individual factors may be unmalleable (e.g., age, gender), and family factors (e.g., parent-child relationships) may be challenging to incorporate into “real-world” intervention efforts (García-Carrión et al., 2019), peer relationships and school environment can be directly targeted in school-based intervention programs.

As a result, the current study uses a social ecological approach to elucidate critical peer and school factors associated with adolescent mental health, while accounting for individual and family characteristics.

### School Climate and School-Based Peer Relationships

Previous research has investigated associations between school characteristics and adolescent mental health, focusing mainly on school demographic factors, including school size, gender composition, and economic deprivation (Saab & Klinger, 2010; Vas et al., 2014). However, in order for research to effectively inform intervention efforts, malleable school-based factors, such as school climate, must also be identified. School climate refers to the larger environmental characteristics of a school, including, but not limited to, values and norms, relationships between teachers and students, and learning and teaching emphasis (Thapa et al., 2013). Recent research suggests that aspects of school climate are associated with adolescent mental health. For example, adolescents who feel connected and safe at school report better mental health (Patalay et al., 2019; Van Ryzin et al., 2009), as do adolescents who perceive their school to be inclusive (László et al., 2019). Supportive teacher-student relationships are associated with better adolescent mental health (Miller-Lewis et al., 2014; Wang et al., 2013). Conversely, school climates that emphasize exam performance have been found to be associated with poor mental health (Byrne et al., 2007; Hogberg et al., 2020). Measurement of school climate has varied across studies, including individual-level perceptions (Van Ryzin et al., 2009), teacher report (László et al., 2019), and collapsing of individual-level reports into aggregate-level measurement (Patalay et al., 2019).

At the same time, adolescence marks a developmental period in which peer relationships grow in salience and become increasingly complex socializing influences (Nelson et al., 2005). Adolescents move from the dyadic friendships that tend to characterize childhood, into larger social networks, often with a heightened emphasis on social position and popularity (Bowker & Ramsay, 2016). Understanding the nature and extent to which peer relationships relate to mental health could offer insight into the design of school-based interventions. To this end, peer relationship quality is known to be associated with mental health, such that supportive friendships are related to positive mental health (Roach, 2018), while peer conflict and victimization by peers is associated with a heightened risk of mental health problems (Patalay & Fitzsimons, 2016). However, the extent to which an adolescent’s popularity with their peers or their friendship network size is related to mental health is currently unknown. Given the changing dynamics of peer relationships during adolescence, and the increased importance of social status during this time (Bowker & Ramsay, 2016), research that includes measures of popularity and friendship quantity would offer new insight into the social context of adolescent mental health.

## Current Study

Taken together, peer relationships and school climate serve as two critically important social ecological domains of influence on adolescent development. Moreover, peer relationships that occur in school, and school factors beyond demographic school-level characteristics (e.g., school climate-related variables), offer potentially modifiable targets for school-based interventions. As such, the primary aim of the current study was to simultaneously estimate the associations between both school-based peer relationships and school climate and adolescent mental health. To shape our analytic approach, we first used methods from multilevel analysis to determine if the nested structure of the data (e.g., students clustered in schools) contributed to differences in mental health. Next, we examined the extent to which peer relationships and school climate were associated with mental health, both independently and in mutually adjusted analyses, also adjusting for background individual and family characteristics.

The study advances previous research by including dimensions of peer relationships related to peer popularity and friendship quantity, in addition to investigating both student-perceived school climate, as well as aggregate-level school climate variables. Based on previous research demonstrating the importance of social status during adolescence (Bowker & Ramsay, 2016; Sweeting & Hunt, 2014), it was expected that peer popularity and friendship quantity would be associated with better mental health. Given evidence of the associations between both individual-perceived and aggregate measures of school climate with mental health (László et al., 2019; Patalay et al., 2019; Van Ryzin et al., 2009), it was expected that: (1) both individual and aggregate measures would be associated when tested independently, but (2) individual-perceptions would mask aggregate effects in mutually adjusted analyses. By investigating multiple dimensions of peer relationships and school climate, the study provides insight into malleable targets that could be leveraged in school-based intervention efforts for adolescent mental health.

## Methods

### Sample

The current study is based on cross-sectional data from 2,571 15–16 year old students in 22 secondary schools (Sweeting et al., 2008), collected in 2006, across a heterogeneous urban area in the West of Scotland. The aim of the full study was to investigate the links between students’ peer group status and levels of stress, and the relationships of these with mental health and health behaviors (see Kelly et al., 2008 for a complete description of the study). Students in all 22 schools completed a questionnaire and took part in a brief interview with trained survey assistants. Previous analyses of this dataset have found associations between the measure of mental health used here and both school disengagement and worry about schools (Sweeting et al., 2010). Informed consent was obtained from adolescents and their parents. The participation rate across the entire sample was 81% (Kelly et al., 2008).

### Measures

#### Mental health

In the current study, adolescent mental health was treated as the dependent variable, and measured using the twelve-item General Health Questionnaire (GHQ-12; Goldberg & Williams, 1988). The GHQ-12 is a widely used tool for screening general psychological distress (Gnambs & Staufenbiel, 2018) validated on adolescents (Tait et al., 2002). In the current study, the GHQ-12 was scored using the Likert method (Goldberg & Williams, 1988), which results in a variable ranging from 0 to 36, with higher scores indicating worse mental health (α = 0.85).

#### Background variables

A range of a priori individual and family characteristic variables were included as predictor variables. *Gender* was a binary variable, based on the question “are you a boy or a girl.” *Ethnicity* was included in the brief interview, in which respondents were provided with a card based on 2001 UK census categories and asked “Which of these groups best describes you?.” Given the homogenous ethnic composition of the sample (see Table 1), a binary variable indicating White versus all other ethnicities was created. *Family affluence* was measured using the Family Affluence Scale (Currie et al., 2008; α = 0.65). This results in a 7-point scale which was collapsed into a 3-point variable of low (0–3), medium (4–5), and high (6–7) affluence. Following previous use of the data (Sweeting et al., 2010), *parental structure* was measured by a binary variable representing those living with both birth parents versus any other situation. The study also included a binary (yes/no) indicator of *longstanding illness*, defined as any illness, disability or infirmity “that has gone on for a long time or is likely to go on for a long time.” To control for associations between *self-esteem* and mental health (Henriksen et al., 2017; Mann et al., 2004), respondents completed a 10-item scale based on Rosenberg’s Self-Esteem Scale (Rosenberg, 1965) with scores ranging from 0 to 30. *Parental control* and *parental care* were measured via the Brief Current Parental Bonding Instrument (Klimidis et al., 1992). Both scales were calculated by summing four ordinal variables (e.g., my parents treat me like a baby; are loving), resulting in scores ranging 0 to 8, with higher scores representing higher levels of control and caring. To control for associations between *family health problems* and adolescent mental health (Moffat & Redmond, 2017), we included a binary (yes/no) variable based on responses to whether “a close family member was seriously ill or injured” within the last year.

**Table 1.** Descriptive Statistics of Sample.

Characteristic	Mean, *SD* or %	Difference between schools (*p*-value)
GHQ-12 Likert score	13.03 (5.40)	.12
*Background variables*		
Age	15.47 (0.32)	<.001
Gender (male)	49.2 %	.07
Ethnicity (white)	92.2 %	<.001
Family affluence	2.29 (0.72)	<.001
Family structure (two parent home)	70.0 %	<.001
Longstanding illness (yes)	10.8 %	.07
Self-esteem	19.81 (4.43)	<.05
Parental control	4.08 (1.54)	<.001
Parental care	8.46 (1.50)	<.05
Family health problems (yes)	35.80 %	<.05
*Peer relationship factors*		
Victimization by school-based peers	1.76 (2.39)	<.001
Self-rated popularity	7.33 (1.82)	<.05
Peer social support	1.31 (0.64)	<.001
Peer popularity	3.76 (2.51)	<.001
Friendship network size	7.83 (3.73)	<.001
*School-based factors*		
School belonging	1.86 (0.65)	<.001
Perceived exam pressure	2.68 (0.50)	.07
School-level exam pressure	2.67 (0.06)	<.001
Student-teacher relationship	1.42 (0.85)	<.001
Perceived inclusivity	1.71 (0.82)	<.001
School-level inclusivity	1.71 (0.13)	<.001

*Note. N* = 2571. Between school differences in sample characteristics were tested with chi-square tests for categorical variables, and ANOVA’s for continuous variables.

*Victimization by school-based peers* was measured by an 8-item scale based on English (Whitney & Smith, 1993) and Irish (O’Moore et al., 1997) studies of bullying. The items asked about frequency of experiences (e.g., physically hurt, hit and kicked, threatened) in school in the current school year, with responses summed to create a continuous variable (range 0–24). *Self-rated popularity* was measured by asking adolescents to indicate, on a 10-rung ladder scale, “how popular you are compared with the rest of your year group.” *Peer social support* was assessed by one item, “I tell my friends about my problems and troubles” (almost always; sometimes; never). *Peer popularity* and *friendship network size* were measured using sociometric data collected in the form of friendship nominations: adolescents were asked to name up to six friends within their school-year group. Peer popularity was measured by the number of incoming friendship nominations an adolescent received, and friendship network size by the summed total of friends they nominated and were nominated by.

#### School climate

School climate was measured through a combination of individual student-perceived and aggregate school-level variables. Individual-level variables were all single items, each asking for agreement (response options: strongly agree; agree; disagree; strongly disagree, scored 0–3, higher scores representing stronger agreement), as follows: s*chool belonging*—“I feel part of this school”; *perceived exam pressure*—“In my school it’s important to pass exams”; *student-teacher relationships*—“When there’s something worrying me there are teachers I can talk to”; *perceived inclusivity*—“In my school it’s OK to be different.” S*chool-level exam pressure* and *school-level inclusivity* variables were created by averaging individual perceived scores per school. Aggregate school-level variables for school belonging and student-teacher relationships were not calculated, given that the survey questions were not worded to reflect school-level perceptions.

### Analyses

Due to the nested structure of the data, with adolescents clustered within schools, preliminary analyses were first conducted to determine whether there were significant between-school differences in GHQ-12 scores, and hence, methods from multilevel modeling would be required. A likelihood ratio test (LRT) was used to compare a model with a multilevel structure (i.e., students nested within schools) to a single-level linear regression model. Results demonstrated that the multilevel model did not fit the data significantly better than the regression model (LRT *x*^2^(1) = 2.79, *p* = .10). In addition, the intraclass correlation coefficient of the multilevel model demonstrated that <1% of the variation in GHQ-12 scores was clustered within schools. Due to the lack of evidence that significant variation in individual mental health scores could be attributed to an adolescent’s school, the remaining analyses used linear regression techniques.

The linear regression models followed a stepwise procedure in order to test the independent and concurrent impact of each social ecological domain, most notably peer and school factors, on adolescent mental health. First, bivariate associations were calculated between each potential explanatory variable and the GHQ-12. Second, a baseline linear regression model including all background variables, representing individual and family characteristics, was conducted (Model 1). Next, two separate regression models were used to test the associations between peer relationships and the GHQ-12 (Model 2), and school climate and the GHQ-12 (Model 3), each adjusted for background variables. Finally, we estimated a model in which variables related to peer relationships and school climate were modeled simultaneously, while accounting for background variables (Model 4).

Nested regression models were compared using the Root Mean Squared Error (RMSE) and the adjusted R^2^. Item-level missingness in the full study sample (N = 3,194) was relatively low, with 4.7% missingness on mental health, and 1 to 5.5% on all other variables. The current study took a complete case approach to missing data, resulting in a final sample size of 2,571. Adolescents were 15.5 years old on average, the sample was split evenly on gender, and the mean GHQ-12 Likert score was 13.1 (range 0–30). See Table 1 for full descriptive statistics for the sample.

## Results

Results for the bivariate associations and nested linear regression models are shown in Table 2. The bivariate analyses showed GHQ-12 scores were higher (i.e., indicating worse mental health) among females, those with a longstanding illness, and those with lower self-esteem. Of the family characteristics, not living with both birth parents, higher parental control, lower parental care, and serious family illness were all associated with higher GHQ-12. In terms of the peer relationship variables, self-rated popularity was negatively associated with GHQ-12, while peer social support, and victimization by peers were positively associated. However, there was no evidence of associations between peer popularity or friendship network size and GHQ-12 scores. All school climate variables, with the exception of school-level inclusivity, showed significant associations with the GHQ-12, with lower scores among those reporting greater school belonging, better relationships with teachers, and higher perceived inclusivity. Conversely, perceived exam pressure and school-level (i.e., aggregate) exam pressure were both associated with higher GHQ-12 scores.

**Table 2.** Results From Final Models.

Parameter	Bivariate	Model 1	Model 2	Model 3	Model 4
	Estimate, *SE*	Estimate, *SE*	Estimate, *SE*	Estimate, *SE*	Estimate, *SE*
Intercept		26.50 (0.81) ***	24.49 (0.87) ***	17.05 (4.21) ***	15.66 (4.17) **
*Background variables*					
Gender	−2.96 (0.14) ***	−1.37 (0.18) ***	−1.52 (0.20) ***	−1.52 (0.18) ***	−1.58 (0.20) ***
Ethnicity	0.47 (0.40)	0.47 (0.32)	0.40 (0.31)	0.49 (0.31)	0.41 (0.32)
Family affluence	−0.09 (0.15)	0.38 (0.12) **	0.27 (0.12) *	0.33 (0.12) **	0.21 (0.12)
Family structure	−1.08 (0.23) ***	−0.26 (0.19)	−0.22 (0.19)	−0.27 (0.19)	−0.23 (0.19)
Long-standing illness	1.10 (0.34) **	0.66 (0.27) **	0.56 (0.27) *	0.71 (0.27) **	0.60 (0.27) *
Self-esteem	−0.66 (0.02) ***	−0.51 (0.02) ***	−0.47 (0.02) ***	−0.49 (0.02) ***	−0.45 (0.02) ***
Parental control	1.00 (0.07) ***	0.36 (0.06) ***	0.31 (0.06) ***	0.34 (0.06) ***	0.30 (0.06) ***
Parental caring	−1.15 (0.07) ***	−0.67 (0.06) ***	−0.62 (0.06) ***	−0.57 (0.06) ***	−0.53 (0.06) ***
Family health problems	1.60 (0.20) ***	0.99 (0.18) ***	0.77 (0.18) ***	0.92 (0.18) ***	0.73 (0.18) ***
*Peer relationship factors*					
Victimization by peers	0.71 (0.04) ***		0.37 (0.04) ***		0.35 (0.04) ***
Self-rated popularity	−0.47 (0.06) ***		0.05 (0.05)		0.03 (0.05)
Peer social support	0.80 (0.17) ***		0.13 (0.15)		0.23 (0.15)
Peer popularity	0.02 (0.04)		0.13 (0.08)		0.01 (0.08)
Friendship network size	−0.07 (0.06)		0.02 (0.06)		0.04 (0.06)
*School climate factors*					
School belonging	−1.87 (0.16) ***			−0.40 (0.15) **	−0.30 (0.15) *
Perceived exam pressure	0.49 (0.21) *			0.55 0.17) **	0.47 (0.17) **
Student-teacher relationship	−1.41 (0.12) ***			−0.49 (0.11) ***	−0.51 (0.11) ***
Perceived inclusivity	−1.01 (0.13) ***			−0.34 (0.11) **	−0.33 (0.11) **
School-level exam pressure	3.62 (1.84) *			2.66 (1.48)	2.65 (1.46)
School-level inclusivity	−0.20 (0.82)			1.04 (0.66)	0.777 (0.65)

*** p < 0.001, **p < 0.01, * p < 0.05.

Results from Model 1, which served as the baseline for the adjusted regression models, demonstrated a range of significant sociodemographic variables. Gender was significantly associated (*b* = −1.37, *p* < .001), with females having higher GHQ-12 scores, representing worse mental health. Those from higher family affluence homes had higher scores (*b* = 0.38, *p* < .05), as did those with a long-standing illness in the family (*b* = 0.85, *p* < .01) and those reporting higher levels of parental control (*b* = 0.36, *p* < .001). Conversely, adolescents with higher self-esteem had lower scores (*b* = −0.51, *p* < .001), as did those reporting higher levels of parental caring (*b* = −0.67, *p* < .001).

Results from Model 2 found mixed evidence for the effect of peer relationships on GHQ-12 scores when adjusted for background variables. Greater victimization by peers was associated with higher scores (*b* = 0.36, *p* < .001). However, peer social support and perceived peer popularity, both of which showed significant associations in the bivariate analyses, were not found to be significant after adjusting for background variables. In addition, there was no evidence of associations between peer popularity or friendship network size and GHQ-12 scores.

Findings from Model 3 demonstrate that multiple aspects of school climate were associated with GHQ-12 scores, after adjusting for background variables. In particular, adolescents who reported a school climate in which it was important to pass exams had higher scores (*b* = 0.55, *p* < .01), while perceiving an inclusive school climate was associated with lower scores (*b* = −0.34, *p* < .01). In addition, adolescents who reported higher levels of school belonging demonstrated lower GHQ-12 scores (*b* = −0.40, *p* < .01), as did those who agreed they had a teacher they could talk to (*b* = −0.54, *p* < .001). Neither aggregate school variable (e.g., school-level exam pressure, school-level inclusivity) was significantly associated with GHQ-12 scores.

Results from Model 4, in which peer relationships and school climate were simultaneously estimated, demonstrated significant associations across both peer and school domains. Adjusted for background variables and school climate, victimization by school-based peers remained the only significant peer relationship variable, with higher levels of victimization associated with higher GHQ-12 scores (*b* = 0.35, *p* < .001). Adjusted for background variables and peer relationship variables, greater school belonging (*b* = −0.30, *p* > 0.05), higher perceived inclusivity (*b* = −0.33, *p* < 0.01), and positive student-teacher relationships (*b* = −0.51, *p* < 0.001) remained associated with lower GHQ-12 scores. Similarly, perceived exam pressure remained associated with higher scores (*b* = 0.47, *p* < .01), while neither school-level aggregate variable (e.g., school-level exam pressure, school-level inclusivity) were significantly associated with GHQ-12 scores. The adjusted *R*^2^ indicates that Model 4 explained 40% of the variation in mental health. The RMSE, a measure of model fit (Pham, 2019), demonstrated that Model 4 was the best-fitting model for our data.

## Discussion

The current study used a social ecological approach to investigate peer relationship and school climate factors associated with adolescent mental health. Importantly, schools have been highlighted as a key context in which adolescent interventions may be carried out (Department of Health and Social Care and the Department of Education, 2017; Stigler et al., 2011), and can feasibly incorporate both peer relationship and school environment components. As such, the study offers critical insight into potential malleable targets for intervention efforts.

Findings from the preliminary multilevel analyses demonstrated no evidence that mental health scores were clustered within schools. This suggests that individual-level variables, rather than school membership, contributed to variation in mental health in the current sample. Results from the linear regression models demonstrated mixed evidence for associations between peer factors and mental health. Adolescents who reported higher levels of victimization by peers in school had worse mental health, while peer support, peer popularity, and friendship network size were not significantly related to mental health outcomes.

The results contrast with previous research that found a protective effect of peer support (Roach, 2018), however the previous findings did not simultaneously include measures of victimization. A supplemental sensitivity analysis on Model 2 revealed a significant and positive association between peer support and mental health when victimization was not included in the model. This suggests that accounting for victimization masks any effect from peer support. Similarly, though self-reported popularity was associated with better mental health in bivariate analyses, this relationship was no longer significant after accounting for additional explanatory variables.

Together, the results demonstrate the importance of peer relationships as a social ecological domain associated with adolescent mental health. In particular, the findings highlight that victimization by peers is a particularly powerful peer experience related to mental health. Thus, results from the current study suggest that targeting healthy peer relationships (e.g., social-emotional learning modules) and specifically striving to combat experiences of peer victimization by school peers (e.g., school policies, skills surrounding bystander interventions) could be useful in universal school-based mental health interventions.

In addition, a range of school climate factors were associated with adolescent mental health. In line with previous research (Miller-Lewis et al., 2014; Patalay et al., 2019; Van Ryzin et al., 2009; Wang et al., 2013), a higher level of school belonging was associated with better mental health, as was positive student-teacher relationships. The positive impact of teachers in the current study supports recent policy work in Scotland regarding the protective effect of “one trusted adult” for adolescent mental health (Whitehead et al., 2019). Further, perceptions of greater exam pressure in school were associated with worse mental health, while those who perceived their school to be inclusive reported better mental health. Both findings agree with previous research (Byrne et al., 2007; Hogberg et al., 2020; László et al., 2019), and provide further evidence that school climate is an important social ecological dimension to consider when designing school-based interventions to support mental health. For example, strategies to alleviate the stress of students in high-pressure academic environments may prove useful, as could incorporating inclusivity (e.g., diversity training) into intervention efforts.

The current study also found that, in the final model, neither aggregate measure of school climate predicted mental health. That is, school-level averages of exam pressure or inclusivity were not significantly associated with individual mental health scores. Supplemental analyses on the final model were conducted to determine whether the simultaneous inclusion of individual-perception and aggregate measures masked individual associations. The models found no significant relationship between school-level exam pressure or school-level inclusivity and mental health, unadjusted for individual-perception of either variable. Although not necessarily objective, school-level aggregate measures reflect the views of the whole year group rather than the individual adolescent. This result is therefore important in suggesting that self-perceived, rather than aggregate levels of these two measures of school climate are associated with mental health.

Despite the many strengths of the study, two primary limitations need to be mentioned. Most notably, the study is cross-sectional in nature, therefore limiting interpretation to associations rather than longitudinal causality. However, by following a step-wise modeling procedure in which social ecological domains are tested independently and concurrently, the study provides a contextualized picture of key factors associated with adolescent mental health. With that in mind, future research should seek to examine changes over time in adolescent mental health in relation to both peer and school characteristics. Second, the data were collected in 2006 and therefore do not include measures related to contemporary adolescent influences, including digital media. Though a limitation, a pre-digital media exploration of peer associations with mental health provides a strong foundation for the relational dimensions of adolescent social life associated with mental health. As such, future research could compare the results of the current study with contemporary data on adolescent mental health in schools.

That said, the current study expands previous research by investigating dimensions of peer relationships related to social status and social networks, as well as concurrently examining individual perceptions of school climate and school-level averages. It highlights that both peer and school climate factors, representing different levels of the social ecological framework, relate to mental health. In particular, the study elucidates potentially modifiable aspects of peer relationships and school climate that could be incorporated into school-based intervention efforts.
